# The Prevalence and Genetic Diversity of PCV3 and PCV2 in Colombia and PCV4 Survey during 2015–2016 and 2018–2019

**DOI:** 10.3390/pathogens11060633

**Published:** 2022-05-31

**Authors:** Diana S. Vargas-Bermudez, José Darío Mogollón, Jairo Jaime

**Affiliations:** Universidad Nacional de Colombia, Sede Bogotá, Facultad de Medicina Veterinaria y de Zootecnia, Departamento de Salud Animal, Centro de Investigación en Infectología e Inmunología Veterinaria (CI3V), Carrera 30 No. 45-03, Bogotá CP 111321, Colombia; dsvargasb@unal.edu.co (D.S.V.-B.); josedmogollon@yahoo.es (J.D.M.)

**Keywords:** porcine circovirus-PCVs, PCV2, PCV3, PCV4, ORF2, prevalence, co-infection

## Abstract

Four genotypes of circovirus have been recognized in swine, with PCV2 and PCV3 being the most associated with clinical manifestations, while PCV4 does not have a defined disease. In addition, PCV2 is associated with different syndromes grouped as diseases associated with porcine circovirus (PCVAD), while PCV3 causes systemic and reproductive diseases. In the present study, we retrospectively detected PCV2, PCV3, and PCV4 in Colombia during two periods: A (2015–2016) and B (2018–2019). During period A, we evaluated stool pools from the 32 Colombian provinces, finding a higher prevalence of PCV3 compared to PCV2 as well as PCV2/PCV3 co-infection. Furthermore, we determined that PCV3 had been circulating since 2015 in Colombia. Regarding period B, we evaluated sera pools and tissues from abortions and stillborn piglets from the five provinces with the highest pig production. The highest prevalence found was for PCV3 in tissues followed by sera pools, while PCV2 was lower and only in sera pools. In addition, PCV2/PCV3 co-infection in sera pools was also found for this period. The complete genome sequences of PCV3 and PCV3-ORF2 placed the Colombian isolates within clade 1 as the majority in the world. For PCV2, the predominant genotype currently in Colombia is PCV2d. Likewise, in some PCV3-ORF2 sequences, a mutation (A24V) was found at the level of the Cap protein, which could be involved in PCV3 immunogenic recognition. Regarding PCV4, retrospective surveillance showed that there is no evidence of the presence of this virus in Colombia.

## 1. Introduction

Circoviruses are non-enveloped and circular single-strained DNA viruses belonging to the Circoviridae family. In pigs, these viruses are widely distributed and are referred to as porcine circoviruses (PCVs). Reviewing the classification of PCVs, it has been evolving as follows: until 2016, PCVs were classified into two types (PCV1 and PCV2), for 2017, the International Committee on Taxonomy of Viruses (ICTV) included a new type called PCV3 [1] and by 2020, PCV4 was reported in China [2]. Reviewing each type, PCV1 was reported in 1974 as a cell culture contaminant and is considered a non-pathogenic virus for swine [3]. PCV2 was discovered in 1998 in Canada and was associated with the postweaning multisystemic wasting syndrome (PMWS) [4]. From this first report, new variants appeared that led to the subclassification of PCV2 in genotypes. For 2008, it was classified into three (PCV2a, PCV2b, and PCV2c); this is supported by the ORF2 sequence [5]. Later, starting in 2010, PCV2d, PCV2e, PCV2f [6,7,8], and PCV2h [9] appeared. Within this broad group of genotypes, it is essential to point out that PCV2a was the most prevalent until 2003, when a genotypic shift was reported, and PCV2b began to predominate. This PCV2b was associated with an increase in both outbreaks and severity of the disease [10]. Then, in 2010, PCV2d appeared and rapidly spread across all continents, displacing and decreasing the prevalence of PCV2b. Currently, PCV2d is considered the most prevalent globally [9]. Regarding the epidemiological behavior of PCV2 genotypes, studies of viral phylodynamics show a cyclical pattern. These cycles consist of several phases: (i) a period of adaptation of the new genotype to naïve pig populations, (ii) an increase in prevalence within those populations, (iii) dissemination through trade routes, (iv) high prevalence worldwide, and (v) finally, the cycle closes when a new emerging genotype replaces the circulating virus [11].

PCV3 was discovered in 2015 in the USA from pigs with varied symptoms such as respiratory failure, multisystemic and cardiac inflammation, reproductive failure, and porcine nephropathy and dermatitis syndrome (PDNS) [12,13]. However, it is essential to point out that, although it was discovered in 2015, retrospective studies show that PCV3 was already present in 1993 in Sweden [14] and 1996 in Spain and China [15,16]. Currently, PCV3 is present on all continents (except Oceania) [13,15,17,18] and if we focus on the Americas, there are reports in the USA [13], Brazil [19], Colombia [20], Chile [21], and Argentina [22]. Regarding clinical manifestations, PCV3 has been found in asymptomatic [16,23,24] and in pigs with symptoms similar to those caused by PCV2, such as PDNS [13], respiratory [25,26], neurological [27], and enteric disorders [28], as well as in reproductive failure, including mummified fetuses, abortion, and stillbirth [13,29,30]. In search of a consensus of symptoms, a classification of the clinical pictures caused by PCV3 into two types was recently proposed: (i) PCV3 reproductive disease (PCV3-RD) and (ii) PCV3 systemic disease (PCV3-SD) [31]. Another important aspect associated with PCV3 is its presence in co-infection with other viruses, such as PCV2 [30,32,33], porcine epidemic diarrhea virus (PEDV) [34], porcine reproductive and respiratory syndrome virus (PRRSV) [35], and classical swine fever virus (CSFV) [35]; as well as with bacteria [25]. Although it has been shown that PCV2 co-infection with other pathogens has led to disease exacerbation [36,37], the impact of PCV3 co-infection with other agents is not yet known [31]. From the evolutionary point of view, unlike PCV2, no significant changes have been reported in PCV3 in terms of sequence [16]. Regarding the latter, when PCV3 appeared, an evolutionary rate (ER) similar to PCV2 of 10^−3^ per site per year (pspy) was estimated. However, recent studies have shown that the rate is lower with approximately 10^−5^ pspy [38]. An additional aspect of PCV3 is that various classifications have been proposed. Initially, it was divided into three genetic groups called PCV3a, PCV3b, and PCV3c based on the amino acid (aa) codons encoded by ORF2 [39]. Subsequently, another classification was proposed in two major groups named a and b (supported on aa motifs in ORF1, ORF2, and ORF3) [40]. Finally, a consensus has been proposed for its classification (based on the whole genome analysis) into clades: clade 1 and clade 2. Thus, clade 1 includes all the PCV3a, PCV3b, and PCV3c genotypes and groups a and b, proposed in the first two classifications, while clade 2 includes some sequences from China [41]. Likewise, the studies that support this classification have revealed no evidence of specific geographic groupings [41].

This study aimed to determine the national prevalence and genetic characteristics of PCV2 and PCV3 in Colombia. Additionally, we performed a PCV4 survey. For the above, different types of samples (serum, stool samples, and tissues from aborted fetuses, and stillborn piglets) were collected in two different periods (2015–2016 and 2018–2019). These samples were evaluated by PCR, sequenced, and analyzed phylogenetically.

## 2. Results

### 2.1. Detection of PCV2, PCV3, PCV4 and Co-Infections between Them

In the present study, we determined the presence of PCV2, PCV3, and PCV4 in Colombia during two different periods: (A) 2015–2016 and (B) 2018–2019. For the first period, we evaluated 755 pools of stools (each pool of five swabs) from the 32 provinces of Colombia (Table 1). Regarding PCV3, we found nine positive pools (nine in 755) corresponding to 1.9%. These positive samples came from six (six in 32) Colombian provinces corresponding to 18.75%. It is essential to point out that two of these provinces (Atlántico and Cundinamarca) are among the five with the highest technical pig production in the country. In the case of PCV2, we found a higher positivity than for PCV3. The positive pools were 69 (69 in 755), corresponding to 9.13%, and they came geographically from 56.25% of the provinces (18 in 32). Four (Antioquia, Atlántico, Cundinamarca, and Risaralda) have the highest technical pig production. When analyzing the simultaneous presence of PCV2 and PCV3 in the same province, we found that it was present in six provinces (18.75%). However, PCV2/PCV3 co-infection (in the same sample) was detected in only one stool pool originating from the province of Atlántico. In order to conduct retrospective surveillance for PCV4 during period A, all samples were tested for this virus. The result was negative (Table 1).

For period B (2018–2019), we evaluated 108 serum pool samples and 19 reproductive tissues (abortions and stillborn piglets) in the five provinces with the highest pig production in Colombia (Table 2). For PCV3, we found a positivity of 43.5% (47 in 108) in pools of sera and all five provinces were positive for PCV3. Regarding the tissues, PCV3 was found in 52.6% (10 in 19). For PCV2, we found a lower prevalence in all provinces evaluated compared to PCV3. In the serum pools, we detected PCV2 in 11% (12 in 108) of the samples and only in two provinces (Atlántico and Cundinamarca). Regarding reproductive tissues, in no case nor any province did we find PCV2. Additionally, we found PCV2/PCV3 co-infection in 6.4% (three in 47) serum pools and in two provinces (Atlántico and Cundinamarca). Likewise, a PCV4 survey was carried out for period B and the result was negative.

### 2.2. Sequence Analysis of PCV3 and PCV2 

**Figure 1:**
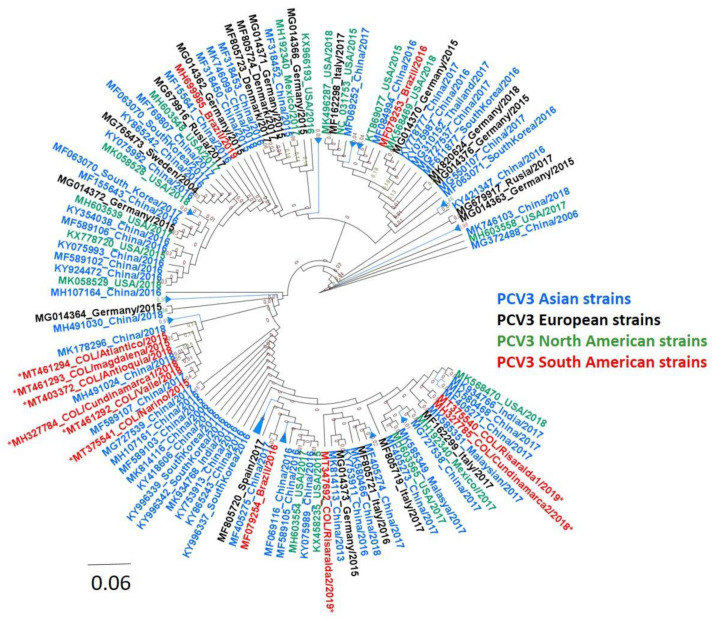
Phylogenetic analysis of PCV3 strains circulating in Colombia during 2015–2019. The phylogenetic tree was constructed by ML analysis using the Tamura-Nei model with gamma distribution with tree topology evaluated with 1000 bootstrap replicates. The sequences generated in this study were labeled in red with *. In addition, the 136 PCV3 complete genomes published in GenBank were included for the phylogenetic analysis.

The present study obtained nine PCV3-complete genome sequences (accession numbers MT375540-41, MT407372, MT461292-94, MT347692, and MH327784-85). Of these, two came from period A, that is, from stool pools, while the remaining seven were obtained from period B (three from reproductive tissues and four from sera pools). Geographically, two sequences came from each of the provinces of Cundinamarca and Risaralda, while one sequence was obtained from Atlántico, Valle del Cauca, Magdalena, Nariño, and Antioquia. The multiple alignments of these nine sequences showed that they shared a nucleotide identity of 99.14–99.9%. Comparing them with 127 PCV3-complete genomes available in the GenBank, we found a nucleotide identity of 97.7–99.74%. The phylogenetic tree built with the 136 strains reported in GenBank showed that the Colombians strains were located in clade 1 associated with others reported in different continents, such as South America, North America, Asia, and Europe (Figure 1). It is important to remember that, to date, clade 2 contains some PCV3 sequences reported only in China. When we analyzed only the PCV3-ORF2 sequence of the nine Colombian strains, we found that all maintained the length of 645 nt encoding to 214 aa capsid protein and shared 98.8–100% nucleotide identity. In addition, we found one aa substitution (A24V) in the Cap protein in three Colombian strains (MT375540-COL/Risaralda1/2018, MT347692-COL/Risaralda2/2019, and MH327785-COL/Cundinamarca2/2018). When we compared the PCV3-ORF2 of our nine sequences with the 127 reported in GenBank, we found an identity of 98.3–100%.

**Figure 2:**
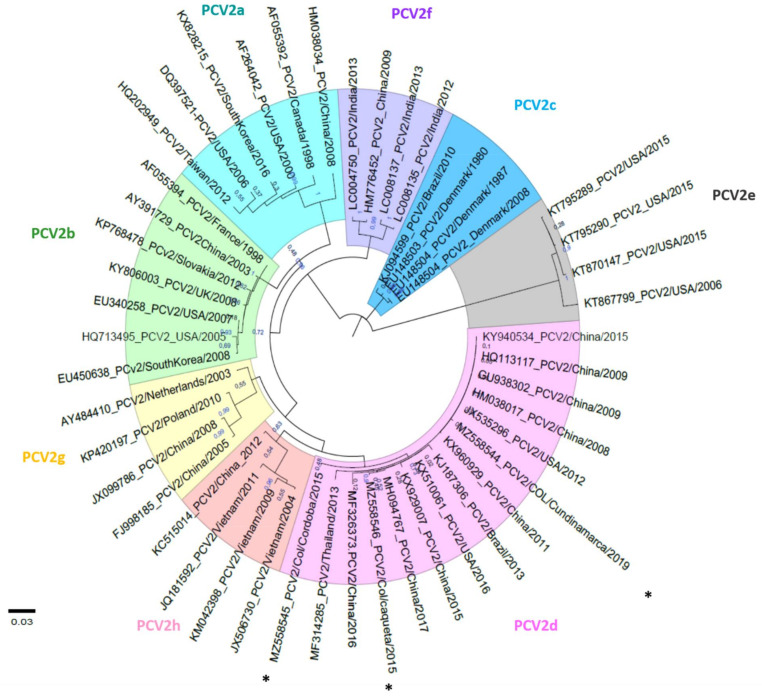
Phylogenetic analysis of PCV2-ORF2 strains circulating in Colombia during 2015–2019. The maximum likelihood phylogenetic tree was constructed using the Tamura Nei model with gamma distribution and tree topology evaluated with 1000 bootstrap replicates. The sequences generated in this study were labeled with *. The 45 PCV2-ORF2 genes published in GenBank were included in the phylogenetic analysis.

Regarding PCV2, we obtained three PCV2-ORF2 sequences (accession number MZ558544-46), two from period A (stool pools from Córdoba and Caquetá) and one from period B (pool of sera from Cundinamarca). Among these three sequences, we find an identity of 99.1–99.3%, while when comparing them with 45 PCV2-ORF2 sequences available in the GenBank, we found 75.4–100% identity. The phylogenetic analysis indicated that the Colombian PCV2 strains sequenced belonged to PCV2d (Figure 2). Regarding the aa sequences encoded by PCV2d-ORF2 (Cap protein), we found a high identity (99.1–99.6%) among the Colombian strains. When comparing these in the two study periods, we found that in period A, the two Cap sequences showed F23, while the one in period B they showed an F23I substitution. Additionally, in all three, we find a G169R substitution. For PCV3-ORF2, a high identity (99.5–100%) was found among the nine Colombian Cap sequences. Between the two periods, we find an A24V substitution. The two Cap proteins sequenced from period A were A24, while in period B, of seven Cap proteins, four maintained A24, and three presented the A24V substitution.

## 3. Discussion

Since first reported, PCVs have been studied to determine the prevalence, characterization, genetic evolution, pathogenesis, and association as a putative pathogen in pigs’ generation of clinical disease. Regarding pig production in Colombia, it is unequally distributed in the 32 provinces of the country. It is carried out technically (farms certified in genetics, health, and biosafety) and informally (traditionally backyard producers and small non-certified farms). Technified production is mainly concentrated in five provinces (Antioquia, Atlántico, Cundinamarca, Risaralda, and Valle). Based on these approaches, the present study sought to determine the behavior of PCV2, PCV3, and PCV4 in Colombia during the last years. For this purpose, we evaluated these viruses during two different periods called A and B.

During period A (2015–2016), we found a low PCV3 prevalence (1.19%) in stool pools from two (two in 32) provinces of Colombia (Atlántico and Cundinamarca). From this result, it is essential to point out that, before this study, the first report of PCV3 in Colombia was made in 2019 [20] from samples of sera and lymphatic nodes. Therefore, we can point out that the virus has already been circulating in Colombia since 2015 and has been maintained particularly in a province (Cundinamarca) with high pig production. This result agrees with other studies [14,15,16], where, retrospectively, it was shown that PCV3 was already circulating before its official report. For Period B (2018–2019), we evaluated sera pools and reproductive tissues (abortions and stillborn piglets) in Colombia’s five provinces with the highest pig production. Although the samples between the two periods were not the same, the prevalence of PCV3 (43.5% in sera pools and 52.6% in reproductive tissues) in period B is striking, indicating that at least in areas of high swine production, the spread of PCV3 was extensive compared to period A. Period B also corresponded with reports of increased reproductive failure in the five provinces studied. In fact, the studies we performed at that time looking for the causality of these reproductive failures initially indicated the presence of PCV3 [20] and the possible participation of this virus as a putative agent of these failures [30]. In this sense, the results of period B (high prevalence in tissues of reproductive origin and low prevalence in stool samples) are consistent with previous studies, where high rates of PCV3 were reported in pigs with reproductive failure [29,35,42,43] and much lower positivity (2.86–6.6%) in stool samples [28,42]. Likewise, in the same period, in reproductive tissue, we found PCV3 and not PCV2, which was also reported by [44], who confirmed that PCV3 is associated with reproductive failure and proposed that PCV2 mainly has a tropism for lymphoid organs.

In the case of PCV2, previous studies reported that the prevalent genotypes in Colombia for 2014 were PCV2b (82%) and PCV2a (18%) [45]. However, our study found that, after 2015, the prevalent genotype was PCV2d. The latter indicates that, in Colombia, this virus has followed an evolutionary pattern similar to that reported in other countries [46]. Comparing the two periods, we found in A a national prevalence of 9.13% in stool pools, being similar (11%) for period B (although with different samples) in the five provinces evaluated. Therefore, regarding PCV2 and its similar and low prevalence during both periods, it is essential to point out as a unifying aspect that in Colombia, we routinely vaccinate against PCV2. Two protocols are applied: the immunization of piglets at weaning and the vaccination in blanket of sows every six months. In the case of this virus, vaccination can reduce viremia even to undetectable levels, as demonstrated in the USA [43], where this routine practice for years generated a decrease in viral load. Additionally, the low prevalence found in our study in stool pools (period A) agrees with those reported by [47], who propose that vaccination against PCV2 has significantly limited viremia and particularly viral elimination through feces. In the case of period B, we found the absence of PCV2 in reproductive tissues that can be explained by the repeated vaccination of pregnant sows, which agrees with [48], who states that this practice generates protection against reproductive infection. In many countries, including Colombia, it is clear that vaccination against PCV2 has resulted in a decrease in prevalence [49]. In addition, previous studies have shown that regardless of the PCV2 vaccine type, the viral load remained low throughout the production cycle [49].

Concerning PCV3/PCV2 co-infection, we found that this was low during the two periods evaluated (11 and 6.4%, respectively). This result agrees with previous studies where PCV3/PCV2 is reported with low prevalences (5.4%) [32] (3.4–8%) [50] and disagrees with others where high prevalences (70%) have been found [34]. The preceding suggests that there is no clear behavior pattern for this co-infection. It can be found without implication in the clinical picture or, on the contrary, that it is associated with it, such as enteric disease, reproductive failure, and respiratory syndrome [34,38,39]. In light of our results and those of others, we can suggest that PCV2 and PCV3 act as individual pathogens during most infections. This approach must be corroborated, hopefully at the field level, determining the presence and involvement of co-infection on specific clinical presentations; Additionally, we must point out that, to date, the impact of this co-infection in pig production systems is unknown.

Another aspect that concerns PCV3 as a topic of discussion is the search for a consensus for the phylogenetic classification. In this sense, there are several proposals [24,25,46] based on the changes of aa at the level of the Cap protein. As stated in the introduction, the current consensus proposal is to divide PCV3 into two clades (1 and 2), taking into account bootstrap support >0.9 and a maximum genetic distance of 3% and 6% at the complete genome and at the ORF2 level [41]. Based on this classification, phylogenetic analyses of the PCV3-complete genome and PCV3-ORF2 sequences presented in this study are located in clade 1, like most strains in the world (except some of China). Additionally, our sequences presented a high identity between them and the strains reported worldwide. The classification into only two clades indicates that PCV3 is a genomically stable virus and that, unlike PCV2, there is no evidence of variants leading to the generation of genotypes. However, it is essential to recommend routine sequencing, particularly of PCV3-ORF2, to establish the evolutionary changes that the virus acquires.

Unlike what was stated in the previous paragraph, PCV2 is a virus in permanent change. In this sense, apart from many genotypes (PCV2a-h), there are changes at the Cap level that can affect the tropism and the immune response against the virus. In the Colombian viruses sequenced in this study, we found in some of these (three in nine strains) an A24V aa substitution in Cap. This aa is located in a potential region of the Cap epitope (by prediction studies) that is subject to positive selection [51]. The above could suggest that the antigenicity of some Colombian strains could be modified with implications that should be analyzed in subsequent studies.

Although there is no evidence of PCV4 in the Americas, we conducted retrospective surveillance in light of this, taking advantage of the samples collected. The results were negative for all samples. It is essential to monitor PCV4 on all continents to monitor how it spreads worldwide. This surveillance is necessary because PCV4 is spreading widely within China [52,53,54,55], and outside of this country, it has been reported in South Korea [56]. As with PCV2 and PCV3, retrospective studies in China showed that PCV4 had been circulating for ten years before its first report [57]. There are few reports on PCV4 detection outside of Asia. Our study agrees with one carried out with tissue and serum samples from Spain and Italy, where PCV4-DNA was not detected [58].

In conclusion, in the present study, we established retrospectively that PCV3 was already in Colombia since 2015 and that by 2018 it was widely distributed in the provinces with the highest swine production. The PCV3-complete genome and PCV3-ORF2 sequences placed the Colombians strains within clade 1. For PCV2, we found that the predominant genotype is PCV2d, displacing PCV2a and PCV2b. Likewise, in some Colombian PCV2-Cap proteins, we found an A24V substitution that could be involved in antigenic recognition. Regarding co-infections, in the two periods studied, PCV2/PCV3 co-infection was found, and it has been increasing. Finally, we found no evidence of the presence of PCV4 in Colombia.

## 4. Materials and Methods

### 4.1. Samples

In the present study, we analyzed pig samples collected during two time periods: (A) 2015–2016, and (B) 2018–2019. These were stored as DNA extractions at −70 °C in the Animal Virology Laboratory of the Facultad de Medicina Veterinaria y de Zootecnia, Universidad Nacional de Colombia, Bogotá. The samples collected during the first period (A) corresponded entirely to 3875 stool samples collected in pools of five units; each pool was worked as a sample (*n* = 775). It is worth noting that these samples were collected at the time (period A) to detect PEDV since Colombia had an outbreak caused by this virus. Additionally, these 775 pool stool samples came from all provinces (*n* = 32) of Colombia. Regarding the second period (B), we collected two types of samples: (i) blood samples (*n* = 540) from piglets to finishing pigs and sows from which pools (*n* = 108) of five sera were prepared; and (ii) reproductive tissues (*n* = 19) from aborted fetuses and stillborn piglets. The samples of this second period came from the five provinces (Antioquia, Atlántico, Cundinamarca, Risaralda, Valle) where the most significant Colombian pig production is found. The origin, type, and the number of samples are summarized in Table 3.

According to the sample type, for period A, the pools of stool samples were resuspended 1:5 in phosphate-buffered saline (PBS) and vortexed for 5 min and centrifuged at 1500× *g* for 10 min at 4 °C and the supernatants were transferred to a 1.5 mL tube. For period B, we centrifuged the blood samples at 3000× *g* for 10 min and then recovered the serum and established pools corresponding to five sera from the same farm. The reproductive tissues (aborted and stillborn piglets) were ground to powder with liquid nitrogen and diluted in three volumes of PBS. Subsequently, they were centrifuged at 1500× *g* for 10 min at 4 °C, and the supernatants were transferred to a 1.5 mL tube. Once the different samples were processed, we proceeded to carry out the extraction of nucleic acids.

### 4.2. Nucleic Acid Extraction and Detection by PCR of PCV2, PCV3 and PCV4

According to the manufacturer’s instructions, DNA and RNA from each sample (stool and sera pools and tissues) were extracted using the High pure nucleic acid kit (Roche^®^, Ref 11858874001, Mannheim, Germany). PCV2, PCV3, and PCV4 were detected by conventional PCR. It is important to note that samples from period A were initially evaluated for PEDV in 2016. Primers for PCV2, PCV3, and PCV4 are presented in Table 4. In brief, reactions were performed in a total volume of 25 µL containing 0.25 µL of Taq polymerase (5 U/µL) (Go taq flexi-Promega^®^, Ref M8295, Madison, WI, USA), 5X Taq buffer (2.5 µL), 2 mM MgCl2, 0.5 mM dNTPs, 1 µL of each primer (20 µM), and 2 µL of extracted DNA. The PCR reactions were performed on a Biorad^®^-DNA (Hercules, CA, USA) thermocycler using a protocol consisting of denaturation at 94 °C for 5 min, followed by 35 cycles including a denaturation at 94 °C for 30 s, annealing at 58 °C for 30 s, and extension at 72 °C for 45 s, with a final extension at 72 °C for 5 min. Positive/negative status for PCV2, PCV3, and PCV4 was determined by the presence of 505, 340, and 391 bp bands, respectively, on 1% agarose gel.

### 4.3. Sequencing of the PCV2-ORF2 and PCV3 Complete Genome

The capsid protein gene (ORF2) of PCV2 was sequenced using specific primers reported by [60]. The PCV3 full genome was amplified using four sets of specific sequencing primers reported by [13]. Reactions were performed in a total volume of 25 µL containing 0.25 µL of Accu*Prime Taq* (5 U/µL) (Invitrogen^®^, Ref 12339-016, Waltham, MA, USA), 1× AccuPrime PCR Buffer I (2.5 µL), 1 µL of each primer (20 µM), and 2 µL of extracted DNA. The PCR reactions were performed on a Biorad^®^-DNA (Hercules, CA, USA) thermocycler using a protocol consisting of denaturation at 94 °C for 2 min, followed by 35 cycles including a denaturation at 94 °C for 30 s, annealing at 58 °C for 30 s, and extension at 68 °C for 1 min. PCR products were directly sequenced in both directions at the commercial sequencing facility SSiGMol (Servicio de Secuenciación y Análisis Molecular, Instituto de Genética, Universidad Nacional de Colombia).

### 4.4. Phylogenetic Analysis

Nine PCV3-complete genome sequences were obtained and aligned to 136 full-length sequences. Additionally, from these sequences, alignment and comparison of PCV3-ORF2 were performed. The selection criterion for these sequences was the location, seeking to compare our sequences with those reported in different countries and continents. For PCV2, three sequences were obtained from ORF2 and aligned with 50 sequences. Here, the selection criteria was the genotype covering from PCV2a to PCV2h. We divided PCV2 genotypes by the following criteria: when the ORF2 genetic distance between them is 0.035 and agrees with the distance observed between viral sequence groups in the phylogenetic trees [5]. All alignments were made with sequences reported in GenBank, and the alignment was achieved by ClustalW using MEGA7 [61]. Phylogenetic analysis was conducted using the maximum-likelihood (ML) method and the Tamura-Nei and discrete Gamma distribution (TN93 + G) model established upon selecting the best-fit model of nucleotide substitution based and Bayesian information criterion (BIC) as implemented in MEGA7 [61]. The robustness of the ML trees was statically evaluated by bootstrap analysis with 1.000 bootstrap samples. All the reference sequence information is listed in Tables S1 (PCV2 reference strains) and S2 (PCV3 reference strains).

## Figures and Tables

**Table 1:** Prevalence of PCV3, PCV2 and PCV4 and co-infections between them during period A of the study (2015–2016) in the 32 provinces of Colombia.

Province	PCV3	PCV2	PCV4	PCV2/PCV3 Co-Infection
Amazonas	0/5 (0) *	1/5 (20) *	0/5 (0) *	0/9 (0) #
Antioquia	0/72 (0)	8/72 (11.1)	0/72 (0)	0/9 (0)
Arauca	0/17 (0)	1/17 (5.88)	0/17 (0)	0/9 (0)
Atlántico	1/7 (14.2)	2/7 (28.5)	0/7 (0)	1/9 (11.1)
Boyacá	0/38 (0)	2/38 (5.26)	0/38 (0)	0/9 (0)
Bolívar	0/35 (0)	4/35 (12.5)	0/35 (0)	0/9 (0)
Caldas	0/16 (0)	3/16 (18.75)	0/16 (0)	0/9 (0)
Caquetá	0/34 (0)	9/34 (26.4)	0/34 (0)	0/9 (0)
Casanare	0/21 (0)	0/21 (0)	0/21 (0)	0/9 (0)
Cauca	2/9 (22.2)	1/9 (11.1)	0/9 (0)	0/9 (0)
Cesar	0/26 (0)	0/26 (0)	0/26 (0)	0/9 (0)
Chocó	0/2 (0)	0/2 (0)	0/2 (0)	0/9 (0)
Córdoba	2/80 (2.5)	4/80 (5)	0/80 (0)	0/9 (0)
Cundinamarca	2/49 (4)	13/49 (26.5)	0/49 (0)	0/9 (0)
Guainía	0/5 (0)	0/5(0)	0/5 (0)	0/9 (0)
Guajira	0/20 (0)	3/20 (15)	0/20 (0)	0/9 (0)
Guaviare	0/5 (0)	0/5 (0)	0/5 (0)	0/9 (0)
Huila	0/26 (0)	0/26 (0)	0/26 (0)	0/9 (0)
Magdalena	0/22 (0)	1/22 (4.5)	0/26 (0)	0/9 (0)
Meta	0/2 (0)	1/2 (50)	0/2 (0)	0/9 (0)
Nariño	1/62 (1.61)	1/62 (1.6)	0/62 (0)	0/9 (0)
Norte Santander	0/72 (0)	5/72 (6.9)	0/72 (0)	0/9 (0)
Putumayo	0/11 (0)	1/11 (9)	0/11 (0)	0/9 (0)
Quindío	0/2 (0)	0/2 (0)	0/2 (0)	0/9 (0)
Risaralda	0/3 (0)	1/3 (33.3)	0/3 (0)	0/9 (0)
San Andrés	1/5 (20)	1/5 (20)	0/5 (0)	0/9 (0)
Santander	0/23 (0)	0/23 (0)	0/23 (0)	0/9 (0)
Sucre	0/32 (0)	6/32 (18.75)	0/32 (0)	0/9 (0)
Tolima	0/34 (0%)	1/34 (2.9)	0/34 (0)	0/9 (0)
Valle	0/9 (0%)	0/9 (0)	0/9 (0)	0/9 (0)
Vaupés	0/6 (0%)	0/6 (0)	0/6 (0)	0/9 (0)
Vichada	0/5 (0%)	0/5 (0)	0/5 (0)	0/9 (0)
Total	9/755 (1.19%)	69/755 (9.13%)	0/755 (0%)	1/9 (11.1%)

* Positive rate/total (%); # Positive PCV2/total positive PCV3 (%).

**Table 2:** Prevalence of PCV3, PCV2, PCV4 and co-infections between them during period B of the study (2018–2019) in the five provinces with the highest technical production of pork in Colombia.

Province	PCV3		PCV2		PCV4		PCV2/PCV3 Co-Infection	
	Sera Pools	Tissues §	Sera Pools	Tissues	Sera Pools	Tissues	Sera Pools	Tissues
Antioquia	3/9 (33) *	2/2 (100) *	0/9 (0) #	0/2 (0) #	0/9 (0) ¤	0/2 (0) ¤	0/47 (0) ¶	0/10 (0) ¶
Atlántico	14/26 (54)	4/5 (80)	5/26 (19)	0/5 (0)	0/26 (0)	0/5 (0)	1/47 (1.7)	0/10 (0)
Cundinamarca	13/32 (41)	2/6 (33)	7/32 (22)	0/6 (0)	0/32 (0)	0/6 (0)	2/47 (3.5)	0/10 (0)
Risaralda	7/24 (29)	2/4 (50)	0/24 (0)	0/4 (0)	0/24 (0)	0/4 (0)	0/47 (0)	0/10 (0)
Valle	10/17 (59)	0/2 (0)	0/17 (0)	0/2 (0)	017 (0)	0/2 (0)	0/47 (0)	0/10 (0)
Total	47/108 (43.5%)	10/19 (52.6%)	12/108 (11.11%)	0/19 (0%)	0/108 (0%)	0/19 (0%)	3/47 (6.38%)	0/10 (0%)

* Positive PCV3/total samples (%); # Positive PCV2/total samples (%); ¤ Positive PCV4/total samples (%); ¶ Positive PCV2/total positive PCV3 (%); § Reproductive tissues (abortions and stillborn piglets).

**Table 3:** Geographical distribution, type, and number of samples evaluated to detect PCV2 and PCV3 in Colombia during two different periods.

	Period A (2015–2016)	Period B (2018–2019)
Geographical origin of the samples	All provinces of the country (*n* = 32)	Provinces with the highest pork production (*n* = 5)
Type of samples	Stool samples (*n* = 3875)/pools of 5 (*n* = 775)	Blood samples (*n* = 540)/pools of 5 (*n* = 108) Reproductive tissues * (*n* = 19)
Total samples	*n* = 775	*n* = 127

* Abortions and stillborn piglets.

**Table 4:** Primers and probes used in the study for the detection of PCV2, PCV3, and PCV4.

Primer Name	Sequence 5′-3′	Product Size	Reference
PCV2F PCV2R	CACATCGAGAAAGCGAAAGGAAC TGCGGGCCAAAAAAGGTACAGTT	505 bp	[59]
PCV3F PCV3R	CCACAGAAGGCGCTATGTC CCGCATAAGGGTCGTCTTG	340 bp	[13]
PCV4F PCV4R	GTTTTTCCCTTCCCCCACATAG ACAGATGCCAATCAGATCTAGGTAC	391 bp	[53]

## Data Availability

The data that supports the findings of this study are available in the supplementary material of this article.

## Supplementary Materials

The following supporting information can be downloaded at: https://www.mdpi.com/article/10.3390/pathogens11060633/s1, Table S1: List of 45 genomic sequences of porcine circovirus 2-PCV2 used in the study. Table S2: List of 127 genomic sequences of PCV3 used in the study.

## References

[1] Lefkowitz E.J., Dempsey D.M., Hendrickson R.C., Orton R.J., Siddell S.G., Smith D.B. (2018). Virus taxonomy: The database of the International Committee on Taxonomy of Viruses (ICTV). Nucleic Acids Res..

[2] Zhang H.-H., Hu W.-Q., Li J.-Y., Liu T.-N., Zhou J.-Y., Opriessnig T., Xiao C.-T. (2020). Novel circovirus species identified in farmed pigs designated as Porcine circovirus 4, Hunan province, China. Transbound. Emerg. Dis..

[3] Tischer I., Mields W., Wolff D., Vagt M., Griem W. (1986). Studies on epidemiology and pathogenicity of porcine circovirus. Arch. Virol..

[4] Ellis J., Hassard L., Clark E., Harding J., Allan G., Willson P., Strokappe J., Martin K., McNeilly F., Meehan B. (1998). Isolation of circovirus from lesions of pigs with postweaning multisystemic wasting syndrome. Can. Vet. J..

[5] Segalés J., Olvera A., Grau-Roma L., Charreyre C., Nauwynck H., Larsen L., Dupont K., McCullough K., Ellis J., Krakowka S. (2008). PCV-2 genotype definition and nomenclature. Vet. Rec..

[6] Guo L.J., Lu Y.H., Wei Y.W., Huang L.P., Liu C.M. (2010). Porcine circovirus type 2 (PCV2): Genetic variation and newly emerging genotypes in China. Virol. J..

[7] Davies B., Wang X., Dvorak C.M.T., Marthaler D., Murtaugh M.P. (2016). Diagnostic phylogenetics reveals a new Porcine circovirus 2 cluster. Virus Res..

[8] Bao F., Mi S., Luo Q., Guo H., Tu C., Zhu G., Gong W. (2018). Retrospective study of porcine circovirus type 2 infection reveals a novel genotype PCV2f. Transbound. Emerg. Dis..

[9] Franzo G., Segalés J. (2018). Porcine circovirus 2 (PCV-2) genotype update and proposal of a new genotyping methodology. PLoS ONE.

[10] Segalés J., Kekarainen T., Cortey M. (2013). The natural history of porcine circovirus type 2: From an inoffensive virus to a devastating swine disease?. Vet. Microbiol..

[11] Franzo G., Cortey M., Segalés J., Hughes J., Drigo M. (2016). Phylodynamic analysis of porcine circovirus type 2 reveals global waves of emerging genotypes and the circulation of recombinant forms. Mol. Phylogenet. Evol..

[12] Phan T.G., Giannitti F., Rossow S., Marthaler D., Knutson T.P., Li L., Deng X., Resende T., Vannucci F., Delwart E. (2016). Detection of a novel circovirus PCV3 in pigs with cardiac and multi-systemic inflammation. Virol. J..

[13] Palinski R., Piñeyro P., Shang P., Yuan F., Guo R., Fang Y., Byers E., Hause B.M. (2017). A Novel Porcine Circovirus Distantly Related to Known Circoviruses Is Associated with Porcine Dermatitis and Nephropathy Syndrome and Reproductive Failure. J. Virol..

[14] Ye X., Berg M., Fossum C., Wallgren P., Blomström A.-L. (2018). Detection and genetic characterisation of porcine circovirus 3 from pigs in Sweden. Virus Genes.

[15] Sun J., Wei L., Lu Z., Mi S., Bao F., Guo H., Tu C., Zhu Y., Gong W. (2018). Retrospective study of porcine circovirus 3 infection in China. Transbound. Emerg. Dis..

[16] Klaumann F., Franzo G., Sohrmann M., Correa-Fiz F., Drigo M., Núñez J.I., Sibila M., Segalés J. (2018). Retrospective detection of Porcine circovirus 3 (PCV-3) in pig serum samples from Spain. Transbound. Emerg. Dis..

[17] Franzo G., Legnardi M., Hjulsager C.K., Klaumann F., Larsen L.E., Segales J., Drigo M. (2018). Full-genome sequencing of porcine circovirus 3 field strains from Denmark, Italy and Spain demonstrates a high within-Europe genetic heterogeneity. Transbound. Emerg. Dis..

[18] Molini U., Marruchella G., Matheus F., Hemberger Y.M., Chiwome B., Khaiseb S., Cattoli G., Franzo G. (2021). Molecular investigation of porcine circovirus type 3 infection in pigs in namibia. Pathogens.

[19] Tochetto C., Lima D.A., Varela A.P.M., Loiko M.R., Paim W.P., Scheffer C.M., Herpich J.I., Cerva C., Schmitd C., Cibulski S.P. (2018). Full-Genome Sequence of Porcine Circovirus type 3 recovered from serum of sows with stillbirths in Brazil. Transbound. Emerg. Dis..

[20] Vargas-Bermudez D.S., Campos F.S., Bonil L., Mogollon D., Jaime J. (2019). First detection of porcine circovirus type 3 in Colombia and the complete genome sequence demonstrates the circulation of PCV3a1 and PCV3a2. Vet. Med. Sci..

[21] Rubilar P.S., Tognarelli J., Fernández J., Valdés C., Broitman F., Mandakovic D., Pulgar R. (2020). Swine viral detection by adapted Next-Generation Sequencing (NGS) for RNA and DNA species reveals first detection of porcine circovirus type 3 (PCV3) in Chile. bioRxiv.

[22] Serena M.S., Cappuccio J.A., Barrales H., Metz G.E., Aspitia C.G., Lozada I., Perfumo C.J., Quiroga M.A., Piñeyro P., Echeverría M.G. (2020). First detection and genetic characterization of porcine circovirus type 3 (PCV3) in Argentina and its association with reproductive failure. Transbound. Emerg. Dis..

[23] Zheng S., Wu X., Zhang L., Xin C., Liu Y., Shi J., Peng Z., Xu S., Fu F., Yu J. (2017). The occurrence of porcine circovirus 3 without clinical infection signs in Shandong Province. Transbound. Emerg. Dis..

[24] Kwon T., Yoo S.J., Park C.-K., Lyoo Y.S. (2017). Prevalence of novel porcine circovirus 3 in Korean pig populations. Vet. Microbiol..

[25] Kim S.-H., Park J.-Y., Jung J.-Y., Kim H.-Y., Park Y.-R., Lee K.-K., Lyoo Y.S., Yeo S.-G., Park C.-K. (2018). Detection and genetic characterization of porcine circovirus 3 from aborted fetuses and pigs with respiratory disease in Korea. J. Vet. Sci..

[26] Kedkovid R., Woonwong Y., Arunorat J., Sirisereewan C., Sangpratum N., Lumyai M., Kesdangsakonwut S., Teankum K., Jittimanee S., Thanawongnuwech R. (2018). Porcine circovirus type 3 (PCV3) infection in grower pigs from a Thai farm suffering from porcine respiratory disease complex (PRDC). Vet. Microbiol..

[27] Chen G.H., Mai K.J., Zhou L., Wu R.T., Tang X.Y., Wu J.L., He L.L., Lan T., Xie Q.M., Sun Y. (2017). Detection and genome sequencing of porcine circovirus 3 in neonatal pigs with congenital tremors in South China. Transbound. Emerg. Dis..

[28] Zhai S.-L., Zhou X., Zhang H., Hause B.M., Lin T., Liu R., Chen Q.-L., Wei W.-K., Lv D.-H., Wen X.-H. (2017). Comparative epidemiology of porcine circovirus type 3 in pigs with different clinical presentations. Virol. J..

[29] Mora-Díaz J., Piñeyro P., Shen H., Schwartz K., Vannucci F., Li G., Arruda B., Giménez-Lirola L. (2020). Isolation of PCV3 from Perinatal and Reproductive Cases of PCV3-Associated Disease and In Vivo Characterization of PCV3 Replication in CD/CD Growing Pigs. Viruses.

[30] Vargas-Bermúdez D.S., Vargas-Pinto M.A., Mogollón J.D., Jaime J. (2021). Field infection of a gilt and its litter demonstrates vertical transmission and effect on reproductive failure caused by porcine circovirus type 3 (PCV3). BMC Vet. Res..

[31] Saporiti V., Franzo G., Sibila M., Segalés J. (2021). Porcine circovirus 3 (PCV-3) as a causal agent of disease in swine and a proposal of PCV-3 associated disease case definition. Transbound. Emerg. Dis..

[32] Wang Y., Noll L., Lu N., Porter E., Stoy C., Zheng W., Liu X., Peddireddi L., Niederwerder M., Bai J. (2020). Genetic diversity and prevalence of porcine circovirus type 3 (PCV3) and type 2 (PCV2) in the Midwest of the USA during 2016-2018. Transbound. Emerg. Dis..

[33] Xia D., Huang L., Xie Y., Zhang X., Wei Y., Liu D., Zhu H., Bian H., Feng L., Liu C. (2019). The prevalence and genetic diversity of porcine circovirus types 2 and 3 in Northeast China from 2015 to 2018. Arch. Virol..

[34] Guo Z., Ruan H., Qiao S., Deng R., Zhang G. (2020). Co-infection status of porcine circoviruses (PCV2 and PCV3) and porcine epidemic diarrhea virus (PEDV) in pigs with watery diarrhea in Henan province, central China. Microb. Pathog..

[35] Chen N., Huang Y., Ye M., Li S., Xiao Y., Cui B., Zhu J. (2019). Co-infection status of classical swine fever virus (CSFV), porcine reproductive and respiratory syndrome virus (PRRSV) and porcine circoviruses (PCV2 and PCV3) in eight regions of China from 2016 to 2018. Infect. Genet. Evol..

[36] Opriessnig T., Gauger P.C., Faaberg K.S., Shen H., Beach N.M., Meng X.-J., Wang C., Halbur P.G. (2012). Effect of porcine circovirus type 2a or 2b on infection kinetics and pathogenicity of two genetically divergent strains of porcine reproductive and respiratory syndrome virus in the conventional pig model. Vet. Microbiol..

[37] Opriessnig T., Halbur P.G. (2012). Concurrent infections are important for expression of porcine circovirus associated disease. Virus Res..

[38] Franzo G., He W., Correa-Fiz F., Li G., Legnardi M., Su S., Segalés J. (2019). A Shift in Porcine Circovirus 3 (PCV-3) History Paradigm: Phylodynamic Analyses Reveal an Ancient Origin and Prolonged Undetected Circulation in the Worldwide Swine Population. Adv. Sci..

[39] Fu X., Fang B., Ma J., Liu Y., Bu D., Zhou P., Wang H., Jia K., Zhang G. (2018). Insights into the epidemic characteristics and evolutionary history of the novel porcine circovirus type 3 in southern China. Transbound. Emerg. Dis..

[40] Fux R., Söckler C., Link E.K., Renken C., Krejci R., Sutter G., Ritzmann M., Eddicks M. (2018). Full genome characterization of porcine circovirus type 3 isolates reveals the existence of two distinct groups of virus strains. Virol. J..

[41] Franzo G., Delwart E., Fux R., Hause B., Su S., Zhou J., Segalés J. (2020). Genotyping Porcine Circovirus 3 (PCV-3) Nowadays: Does It Make Sense?. Viruses.

[42] Saporiti V., Cruz T.F., Correa-Fiz F., Núñez J.I., Sibila M., Segalés J. (2020). Similar frequency of Porcine circovirus 3 (PCV-3) detection in serum samples of pigs affected by digestive or respiratory disorders and age-matched clinically healthy pigs. Transbound. Emerg. Dis..

[43] Dvorak C.M.T., Yang Y., Haley C., Sharma N., Murtaugh M.P. (2016). National reduction in porcine circovirus type 2 prevalence following introduction of vaccination. Vet. Microbiol..

[44] Segalés J., Mateu E. (2006). Immunosuppression as a feature of postweaning multisystemic wasting syndrome. Vet. J..

[45] Monroy M.A.R., Ramirez-Nieto G.C., Vera V.J., Correa J.J., Mogollón-Galvis J.D. (2014). Detection and molecular characterization of porcine circovirus type 2 from piglets with porcine circovirus associated diseases in Colombia. Virol. J..

[46] Xiao C.-T., Harmon K.M., Halbur P.G., Opriessnig T. (2016). PCV2d-2 is the predominant type of PCV2 DNA in pig samples collected in the U.S. during 2014-2016. Vet. Microbiol..

[47] Woźniak A., Miłek D., Matyba P., Stadejek T. (2019). Real-Time PCR Detection Patterns of Porcine Circovirus Type 2 (PCV2) in Polish Farms with Different Statuses of Vaccination against PCV2. Viruses.

[48] Pejsak Z., Kusior G., Pomorska-Mól M., Podgórska K. (2012). Influence of long-term vaccination of a breeding herd of pigs against PCV2 on reproductive parameters. Pol. J. Vet. Sci..

[49] Vargas-Bermudez D.S., Díaz A., Mogollón J.D., Jaime J. (2018). Longitudinal comparison of the humoral immune response and viral load of Porcine Circovirus Type 2 in pigs with different vaccination schemes under field conditions. F1000Research.

[50] Jiang H., Wang D., Wang J., Zhu S., She R., Ren X., Tian J., Quan R., Hou L., Li Z. (2019). Induction of Porcine Dermatitis and Nephropathy Syndrome in Piglets by Infection with Porcine Circovirus Type 3. J. Virol..

[51] Li G., He W., Zhu H., Bi Y., Wang R., Xing G., Zhang C., Zhou J., Yuen K.-Y., Gao G.F. (2018). Origin, genetic diversity, and evolutionary dynamics of novel porcine circovirus 3. Adv. Sci..

[52] Sun W., Du Q., Han Z., Bi J., Lan T., Wang W., Zheng M. (2021). Detection and genetic characterization of porcine circovirus 4 (PCV4) in Guangxi, China. Gene.

[53] Tian R.-B., Zhao Y., Cui J.-T., Zheng H.-H., Xu T., Hou C.-Y., Wang Z.-Y., Li X.-S., Zheng L.-L., Chen H.-Y. (2021). Molecular detection and phylogenetic analysis of Porcine circovirus 4 in Henan and Shanxi Provinces of China. Transbound. Emerg. Dis..

[54] Ha Z., Yu C., Xie C., Wang G., Zhang Y., Hao P., Li J., Li Z., Li Y., Rong F. (2021). Retrospective surveillance of porcine circovirus 4 in pigs in Inner Mongolia, China, from 2016 to 2018. Arch. Virol..

[55] Chen N., Xiao Y., Li X., Li S., Xie N., Yan X., Li X., Zhu J. (2020). Development and application of a quadruplex real-time PCR assay for differential detection of porcine circoviruses (PCV1 to PCV4) in Jiangsu province of China from 2016 to 2020. Transbound. Emerg. Dis..

[56] Nguyen V.-G., Do H.-Q., Huynh T.-M.-L., Park Y.-H., Park B.-K., Chung H.-C. (2021). Molecular based detection, genetic characterization and phylogenetic analysis of porcine circovirus 4 from Korean domestic swine farms. Transbound. Emerg. Dis..

[57] Hou C.-Y., Zhang L.-H., Zhang Y.-H., Cui J.-T., Zhao L., Zheng L.-L., Chen H.-Y. (2021). Phylogenetic analysis of porcine circoviruses 4 in Henan Province of China: A retrospective study from 2011 to 2021. Transbound. Emerg. Dis..

[58] Franzo G., Ruiz A., Grassi L., Sibila M., Drigo M., Segalés J. (2020). Lack of Porcine circovirus 4 Genome Detection in Pig Samples from Italy and Spain. Pathogens.

[59] Yang K., Jiao Z., Zhou D., Guo R., Duan Z., Tian Y. (2019). Development of a multiplex PCR to detect and discriminate porcine circoviruses in clinical specimens. BMC Infect. Dis..

[60] Oliver-Ferrando S., Segalés J., López-Soria S., Callén A., Merdy O., Joisel F., Sibila M. (2016). Evaluation of natural porcine circovirus type 2 (PCV2) subclinical infection and seroconversion dynamics in piglets vaccinated at different ages. Vet. Res..

[61] Kumar S., Stecher G., Tamura K. (2016). MEGA7: Molecular evolutionary genetics analysis version 7.0 for bigger datasets. Mol. Biol. Evol..

