# Supplementary material for: The Prevalence and Genetic Diversity of PCV3 and PCV2 in Colombia and PCV4 Survey during 2015–2016 and 2018–2019
Source: Pathogens. 2022 May 31;11(6):633. doi: 10.3390/pathogens11060633 (PMC9228467; doi:10.3390/pathogens11060633)
Supplement: Supplementary file 1 [file pathogens-11-00633-s001.zip › pathogens-1689474-Table S1.pdf]

Table S1. List of 45 genomic sequences of porcine circovirus 2-PCV2 used in the study

**Number**	**Genbank**	**Country**	**Collection year**
1	KJ187306	Brazil	2013
2	JX535296	USA	2012
3	HM038017	China	2008
4	AY484410	Netherlands	2003
5	AF055394	France	1998
6	EU340258	USA	2007
7	AF055392	Canada	1998
8	AF264042	USA	2000
9	HM038034	China	2008
10	HQ202949	Taiwan	2012
11	KX828215	SouthKorea	2016
12	KP768478	Slovakia	2012
13	EU450638	SouthKorea	2008
14	KY806003	UK	2008
15	KJ094599	Brazil	2010
16	EU148503	Denmark	1980
17	KC515014	China	2012
18	MF314285	Thailand	2013
19	KX960929	China	2011
20	KT867799	USA	2006
21	KT870147	USA	2015
22	KT795289	USA	2015
23	LC004750	India	2013
24	LC008137	India	2013
25	LC008135	India	2012
26	KP420197	Poland	2010
27	FJ998185	China	2005
28	JX099786	China	2008
29	JQ181592	Vietnam	2011
30	JX506730	Vietnam	2004
31	KM042398	Vietnam	2009
32	EU148504	Denmark	1987
33	GU938302	China	2009
34	KY940534	China	2015
35	HQ113117	China	2009
36	AY391729	China	2003
37	DQ397521	USA	2006
38	EU148504	Denmark	2008
39	HM776452	China	2009

40	HQ713495	USA	2005
41	KT795290	USA	2015
42	KX510061	USA	2016
43	KX929007	China	2015
44	MF326373	China	2016
45	MH094767	China	2017
