# Supplementary material for: The Prevalence and Genetic Diversity of PCV3 and PCV2 in Colombia and PCV4 Survey during 2015–2016 and 2018–2019
Source: Pathogens. 2022 May 31;11(6):633. doi: 10.3390/pathogens11060633 (PMC9228467; doi:10.3390/pathogens11060633)
Supplement: Supplementary file 1 [file pathogens-11-00633-s001.zip › pathogens-1689474-Table S2.pdf]

Table S2. List of 127 genomic sequences of PCV3 used in the study

Number	Genbank	Country	Collection year
1	MG765473	Sweden	2004
2	MG372488	China	2006
3	MK814116	China	2006
4	MK814113	China	2013
5	MK814114	China	2013
6	KT869077	USA	2015
7	KX458235	USA	2015
8	KX778720	USA	2015
9	NC031753	USA	2015
10	MF589104	Country	2015
11	MF589103	China	2015
12	MF318449	China	2015
13	MF318450	China	2015
14	MF318451	China	2015
15	MF318452	China	2015
16	MG014366	Germany	2015
17	MG014362	Germany	2015
18	MG014363	Germany	2015
19	MG014364	Germany	2015
20	MG014370	Germany	2015
21	MG014371	Germany	2015
22	MG014372	Germany	2015
23	MG014373	Germany	2015
24	MG014376	Germany	2015
25	KX966193	USA	2016
26	MF079253	Brazil	2016
27	MF079254	Brazil	2016
28	KY075992	China	2016
29	KY075993	China	2016
30	KY075994	China	2016
31	MF069116	China	2016
32	KY354038	China	2016
33	KY354039	China	2016
34	KY418606	China	2016
35	KY421347	China	2016
36	KY421348	China	2016
37	KY753911	China	2016
38	MG564174	China	2016
39	MG564175	China	2016

40	KY753912	China	2016
41	KY753913	China	2016
42	KY865242	China	2016
43	KY865243	China	2016
44	KY924472	China	2016
45	KY924473	China	2016
46	KY075986	China	2016
47	KY075987	China	2016
48	KY075988	China	2016
49	KY075990	China	2016
50	KY075989	China	2016
51	MH107161	China	2016
52	MH107164	China	2016
53	MF318448	China	2016
54	MF318453	China	2016
55	MF084994	China	2016
56	MF155641	China	2016
57	MF155643	China	2016
58	MF589102	China	2016
59	MF589105	China	2016
60	MF589106	China	2016
61	KY996337	South Korea	2016
62	KY996338	South Korea	2016
63	KY996339	South Korea	2016
64	KY996341	South Korea	2016
65	KY996342	South Korea	2016
66	KY996343	South Korea	2016
67	KY996344	South Korea	2016
68	MF063071	South Korea	2016
69	MF611876	South Korea	2016
70	MF611877	South Korea	2016
71	MF805721	Italy	2016
72	MH603548	USA	2017
73	MH603554	USA	2017
74	MH603539	USA	2017
75	MH603558	USA	2017
76	MH603565	USA	2017
77	MH192340	Mexico	2017
78	MH192340	Mexico	2017
79	MK585351	Malaysian	2017
80	MK585351	Malaysia	2017

81	MK585349	Malasya	2017
82	MG310152	Thailand	2017
83	MK934767	India	2017
84	MK934766	India	2017
85	MK580468	China	2017
86	MG778698	China	2017
87	MF769807	China	2017
88	MF069252	China	2017
89	KY924475	China	2017
90	KY778777	China	2017
91	MF405271	China	2017
92	MF405272	China	2017
93	MF405273	China	2017
94	MF405274	China	2017
95	MF405275	China	2017
96	MF405276	China	2017
97	MF589107	China	2017
98	MF405277	China	2017
99	MG550107	China	2017
100	MG727537	China	2017
101	MG727538	China	2017
102	MG727539	China	2017
103	MG727540	China	2017
104	MF063070	South Korea	2017
105	MG679916	Rusia	2017
106	MG679917	Rusia	2017
107	MF162298	Italy	2017
108	MF162299	Italy	2017
109	MF805719	Italy	2017
110	MF805720	Spain	2017
111	MF805723	Denmark	2017
112	MF805724	Denmark	2017
113	MK568469	USA	2018
114	MK568470	USA	2018
115	MK058528	USA	2018
116	MK496287	USA	2018
117	MK058529	USA	2018
118	MK746103	China	2018
119	MH491030	China	2018
120	MH491024	China	2018
121	MK178296	China	2018

122	MH491029	China	2018
123	MK580466	China	2018
124	MK746099	China	2018
125	MK934768	India	2018
126	MK820624	Germany	2018
127	MH699985	Brazil	2019
